# Identification of drought-tolerant hub genes in Iranian KC-2226 genotype of *Aegilops tauschii* using transcriptomic analysis

**DOI:** 10.1038/s41598-023-36133-0

**Published:** 2023-06-12

**Authors:** Keyvan Hasanpour, Ali Aalami, Rahele Ghanbari Moheb Seraj, Ramin Hosseini, Shahram Naeimi, Keyvan Esmaeilzadeh-Salestani

**Affiliations:** 1 Department of Agricultural Biotechnology, University of Guilan, University Campus 2, Rasht, Iran; 2 Department of Agricultural Biotechnology, Faculty of Agricultural Sciences, University of Guilan, Rasht, Iran; 3 Department of Horticultural Sciences, Faculty of Agriculture and Natural Resources, University of Mohaghegh Ardabili, Ardabil, Iran; 4 Department of Biotechnology, Faculty of Agriculture and Natural Resource, Imam Khomeini International University, Qazvin, Iran; 5 Department of Biological Control Research, Iranian Research Institute of Plant Protection, Agricultural Research, Education and Extension Organization (AREEO), Tehran, 19858-13111 Iran; 6 Chair of Crop Science and Plant Biology, Institute of Agricultural and Environmental Sciences, Estonian University of Life Sciences, Kreutzwaldi 1, 51006 Tartu, Estonia

**Keywords:** Biotechnology, Plant sciences

## Abstract

*Aegilops tauschii*, as a donor of D genome to the bread wheat with a valuable source of resistance to different biotic and abiotic stresses, is used to improve the quality of wheat cultivars. Every genotype has a specific genetic content, the investigation of which can lead to the identification of useful genes such as stress tolerance genes, including drought. Therefore, 23 genotypes of *Ae. tauschii* were selected to evaluate their morphological and physiological traits under greenhouse conditions. Among them, a superior tolerant genotype (KC-2226) was chosen for transcriptomic analysis. Our result showed that 5007 and 3489 genes were deferentially up- and downregulated, respectively. Upregulated genes were involved in photosynthesis, glycolysis/gluconeogenesis, and amino acid biosynthesis whereas downregulated genes were often engaged in DNA synthesis, replication, repair and topological changes. The result of protein–protein interaction network analysis showed that AT1G76550 (1.46), AT1G20950 (1.42), IAR4 (1.19), and PYD2 (1.16) among upregulated genes and THY-1 (44), PCNA1 (41) and TOPII (22) among down-regulated genes had the highest interactions with other genes. In conclusion, *Ae. tauschii* employs elevated transcription of specific genes involved in photosynthesis, glycolysis and gluconeogenesis and amino acid biosynthesis pathways rather than genes active in DNA synthesis and repair to provide the energy needed for the plant to survive under stress conditions.

## Introduction

Tausch's goatgrass or rough-spike hard grass (*Aegilops tauschii*) is one of the most advantageous wild species of the Triticeae tribe to improve wheat quality^1,2^. *Ae. tauschii* Coss. (2n = 2x = 14) is a donor of D genome to cultivated wheat (*Triticum aestivum* L = AABBDD, 2n = 42, hexaploid)^3,4^. The DD genome includes genes engaged in resistance to different biotic and abiotic stresses, which provides the potential for facilitating the improvement of wheat cultivars^5^. Wheat as an important strategic crop is placed at the center of human food and nutrition^6^. The world population is rapidly growing and this highlights the importance of producing more foods, especially wheat, to meet the quickly increasing demand and ensure food security. Therefore, producing wheat of those cultivars that are tolerant to stress, particularly drought when we are facing global warming, is of prime importance.

Biotic and abiotic stresses including drought affect the quality and yield of wheat, leading the research toward the understanding of the molecular mechanism of plant responses to stress^7–10^. Plants adopt different cellular responses including stomatal closure, activating transcription, changing the membrane stability, accumulating osmoprotectants and antioxidants, and scavenging reactive oxygen species (ROS) under drought stress^11,12^. These processes are regulated by the expression of different genes at the transcription level.

In our previous study, 23 genotypes of *Ae. tauschii* were examined in terms of morphological, physiological and antioxidant enzymatic activity analysis under drought stress conditions^13^. In the present study, different genotypes were compared and finally the genotype KC-2226 was selected as the most tolerant genotype. For further confirmation, transcriptomics analysis was performed on this genotype. RNA-seq is an efficient procedure to identify differentially expressed genes (DEGs) in a regulatory network at the transcriptome level^14^. Although different transcriptomic studies of *Ae. tauschii* were performed through RNA-Seq analysis under different conditions^15–18^, our study can elucidate molecular mechanisms of plant response to drought stress by identifying hub genes, which could be used for breeding purposes.

## Materials and methods

### Plant material and growth condition

The seeds of nineteen Iranian genotypes of *Ae. tauschii* were obtained from the National Plant Gene Bank of Iran (NPGBI), Seed and Plant Improvement Institute, Karaj, Iran. The seeds of the four remaining genotypes were provided by Ilam University Gene Bank (IUGB), Ilam University, Ilam, Iran (supplementary file 1). The genotype code, Accusable Gene Bank, WIEWS inst. code, country, province, and location of all genotypes were provided in Supplementary file 1. Information about each genotype is available at the National Seed Bank of Iran (Seed and Plant Improvement Institute). Systematic identification of the plant materials was performed in NPGBI. In addition to seed, herbarium specimens of under-studied accessions also are available in NPGBI. Plant studies in this experiment comply with relevant institutional, national, and international guidelines and legislation. The required permits for seed preparation and research on it have been obtained from Guilan University.

The seeds were soaked in water for 3–4 days. Five-six seeds with the same size were sown in each plastic pot (a diameter of 20 cm and a height of 30 cm), containing the same volumes of sandy loam soil. All pots were irrigated every three days and the moisture of the soil was kept at 80–100% field capacity for seed germination. All pots were placed in a greenhouse with a 16/8 (light/dark) photoperiod at 26 ± 2 °C in Imam Khomeini International University, Qazvin, Iran (IKIU).

### Drought stress treatment

The plants were exposed to drought stress 10 days after germination when the seedlings were at the 3–4 leaf stage (BBCH = 13). To treat seedlings with drought stress, they were not irrigated for 14 days whereas the control group received water as before. Soil moisture was measured by using a moisture meter device (Delta-T devices, UK). After finishing exposure of plants to drought stress, the seedlings were collected and their morphological and physiological parameters were analyzed.

### Selection of tolerant genotype

To select the best tolerant genotypes, morphological and physiological traits of *Ae. tauschii*, including relative water content (RWC), root and shoot length of seedlings, the number of leaves and tillers, shoot fresh and dry weight of seedlings, root fresh and dry weight of seedlings, dry weight of mature plant root and shoot, length of flag leaf, peduncle and spikes, and percentage of healthy leaves, enzymatic (catalase, superoxide dismutase, ascorbate peroxidase, and peroxidase) activity, proline and chlorophyll content were investigated in all *Ae. tauschii* genotypes. After analysis of variance, the genotypes were compared based on the mean comparison of traits calculated by Duncan's multiple-range test (p < 0.01) and then ranked according to the method of Arunachalam and Bandyopadhyay^19^.

### RNA extraction and sequencing

Total RNA was extracted from 200 mg ground leaves samples (4 leaf stage) using RNeasy Plant Mini Kit (Qiagen, Germany) according to the manufacturer’s protocol. The quantity and quality of the extracted RNA were analyzed using NanoDrop 1000 spectrophotometer (Thermo Scientific, USA) and 1% agarose gel electrophoresis, respectively. The extracted RNA samples were treated with DNase I (Thermo Scientific, USA) to eliminate probable genomic contamination. Each of the control and drought stress treatments had three replications. Subsequent quality control of the extracted RNA was done using a QC Bioanalyzer (Agilent Technologies, CA, USA). The Poly-A selection, preparation of cDNA, adapter ligation, clusters formation, and sequencing was carried out at Beijing Genomes Institute following the manufacturer’s instruction, using TruSeq Stranded total RNA with Ribo-Zero Plant kit (Illumina, USA). The sequencing was performed by Poly-A mRNA Capture method using a Nova-seq 6000 platform to produce 6 GB raw data of 100 bp paired-end reads.

### Bioinformatics analysis

The quality of sequenced raw reads was evaluated using FastQC software (version 0.11.9)^20^. Trimmomatic (Version 0.36)^21^ was used to remove adaptors and low-quality nucleotides and sequences. The trimmed reads were double-checked using FastQC to verify the efficiency of trimming. Clean high-quality trimmed reads were mapped to a reference genome (https://ftp.ncbi.nlm.nih.gov/genomes/all/GCF/002/575/655/GCF_002575655.2_Aet_v5.0/GCF_002575655.2_Aet_v5.0_genomic.fna.gz) downloaded from NCBI database using HISAT2 (Version 2.2.0)^22^. Reads mapped to each gene were counted by HTSeq (version 2.0.2)^23^ using gene annotation file (https://ftp.ncbi.nlm.nih.gov/genomes/all/GCF/002/575/655/GCF_002575655.2_Aet_v5.0/GCF_002575655.2_Aet_v5.0_genomic.gff.gz) downloaded from NCBI database. Differentially expressed genes (DEGs) were identified by DESeq2 R package (version 4.2)^24^ with a threshold of the adjusted p-value (p.adj) ≤ 0.01 and an absolute value of log2FoldChange ≥  ± 2. The GO enrichment analysis of DEGs was carried out using AmiGO (version 2.0)^25^. The KAAS (KEGG Automatic Annotation Server) database^26^ was used to identify enriched DEGs in KEGG pathways. Fisher’s exact test and p.adj ≤ 0.01 were used to identify significant pathways. Volcano plot was created using VolcaNoseR web app (https://huygens.science.uva.nl/VolcaNoseR/)^27^. Bubble and Butterfly bar plots were constructed by SRplot (http://www.bioinformatics.com.cn/en). Protein–protein interaction (PPI) network was determined by STRING database^28^, using *Arabidopsis thaliana* as model plant and then imported into Cytoscape software (version 3.9.1)^29^ for visualization and edition. The gene network was plotted using the CytoHubba plugin^30^ based on three MCC algorithm.

### Expression analysis and validation of RNA-seq data

Purified RNA (Concentration 1 µg, from section "RNA extraction and sequencing") was used to synthesize first-strand cDNA through cDNA Synthesis kit (RB125A, RNA, Iran) following the manufacturer’s protocol. To validate the expression of selected hub genes acquired from RNA-seq in *Ae. tauschii*, an RT-qPCR analysis was performed for two up- and two downregulated genes. To design primers of the genes of interest, nucleotide sequences of genes were obtained from the sequencing data. Specific primers were designed from areas near the end of poly adenine, with a length of 150–250 bp. Homodimer, heterodimer, stem-loop, GC percent, and TM temperature were measured using Oligo 7 Primer Analysis Software (v7.60) (https://www.oligo.net/)^31^ and Vector NTI^®^ Express Designer Software (v11.0) (https://vector-nti.software.informer.com/11.0/)^32^. Finally, primers were produced by Bioneer Company (South Korea). The sequence and other information of primers are listed in Table 1. The *Actin* gene was used as a reliable reference gene. RT-qPCR amplification was carried out by Rotor-Gene 2000 (Corbett Life Science, Sydney, Australia) using SYBR^®^ Green Real-Time PCR Master Mix (RB120, RNA, Iran). The thermal conditions consisted of an initial step for 5 min at 95 °C, followed by 35 cycles amplification (1 min at 95 °C, 1 min at 50–60 °C depending on primers tm, and 15 s at 72 °C). To investigate the specificity of each amplicon, post-amplification melting-curve ranging from 60 to 95 °C were assessed in every reaction. The relative expression of genes was calculated by REST software (v2009) (https://www.gene-quantification.de/rest-2009.html) with the following formula. The E in the equation refers to the primer efficiency.

$$ Gene\;expression\;ratio = \frac{{(E_{Gene} )^{\Delta ct\;Gene} }}{{(E_{Ref} )^{\Delta ct\;Ref} }} $$

**Table 1:** RT-qPCR primer sequence of selected hub and reference genes and their amplification characteristics.

Primer name	Primer sequence	PCR product length (bp)	TM	PCR amplification efficiency
*IAR4*	F: GGTCCTATTATCCTTGAGATGG	224	48.9	1.92
	R: GTGAATAGCTCAGATGCATC		44.3	
*PYD2*	F: GTGGAGACCGGCAAGATCAC	152	54.2	1.89
	R: CACGTTCGTGTCGGACCTCG		58.6	
*TOPII*	F: GAGCAAAGAAGAAGGCTCCAG	257	56.3	1.96
	R: CACACCAATTGCCAGAACCTC		54.3	
*THY-1*	F: TATCAGCGTTCAGCAGATATG	238	48.2	1.90
	R: CTTGTGAGGATCATAGCCTG		47	
	R: CTTCTGGAAATGCTAATC		49	
*Actin*	F: CCAGGGCAGAGTACGAAGAG	153	51.5	1.96
	R: GGAACATGGTAGACCCACC		55	

## Results

### Selection of tolerant genotype

**Figure 1:**
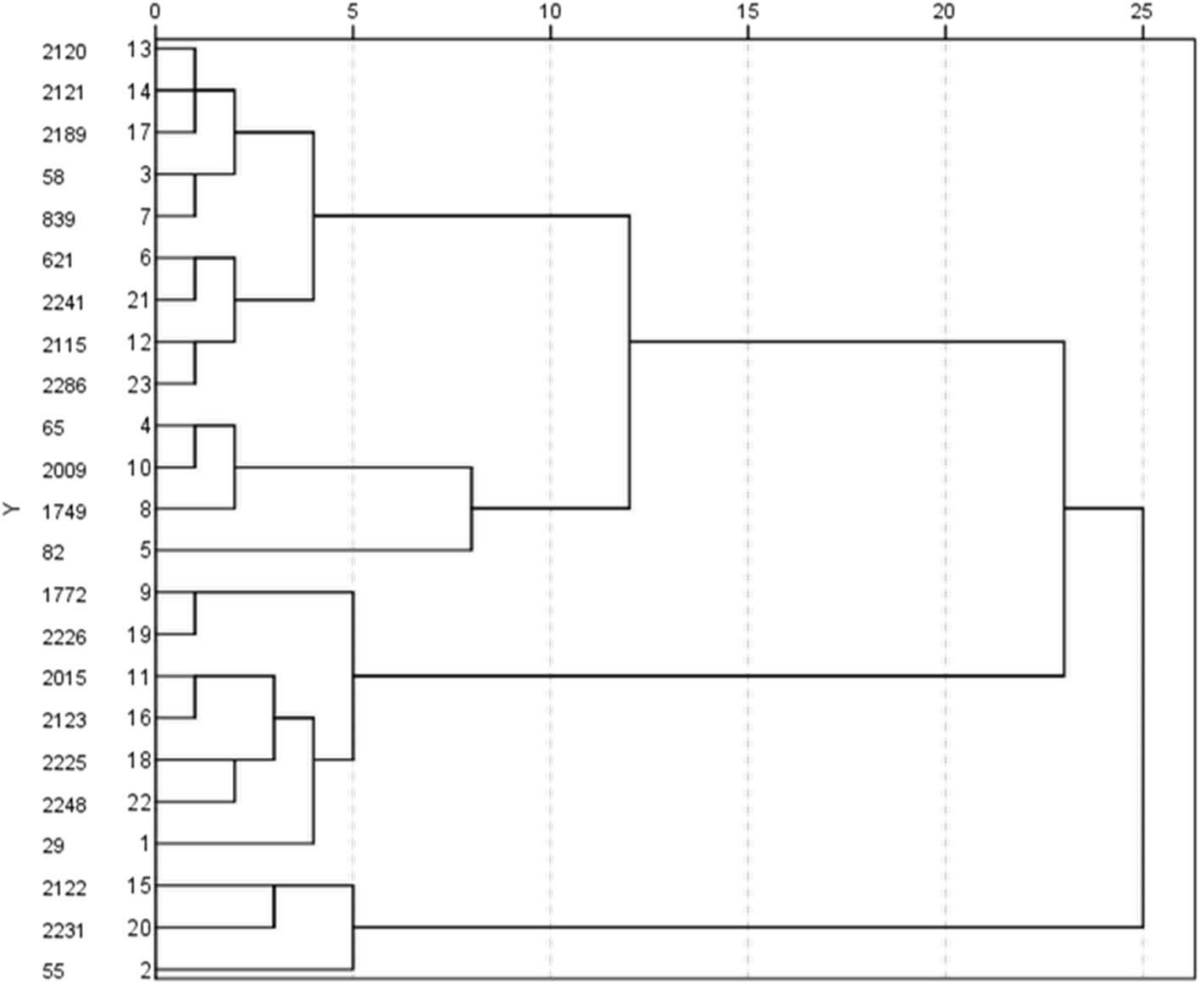
Clustering of 23 studied genotypes at seedling and maturity growth stage. Horizontal axis indicated genotypes distance in terms of all the examined traits. Vertical axis represented 23 genotypes of *Ae. tauschii*.

According to Table 2, all 23 genotypes of *Ae. tauschii* were compared in terms of morphological and physiological traits under drought stress conditions. First, analysis of variance and mean values for all traits were calculated. Next, the rank of all genotypes was determined based on the mean values of traits. Based on Arunachalam and Bandyopadhyay method, the genotypes with lower ranks have a higher mean of analyzed traits. Therefore, genotypes KC-2231, KC-2225 and KC-2226 were selected as tolerant genotypes to drought (Table 2). On the other hand, cluster analysis revealed that genotypes KC-2225 and KC-2226 were placed into group 3, indicating having a higher mean of studied traits (Fig. 1). Finally, based on the above two analyses, genotype KC-2226 was selected for transcriptome analysis.

**Table2:** Mean comparison and Arunachalam and Bandyopadhyay ranking of morpho- and physiological traits of *Ae. tauschii* genotypes under drought stress.

Genotypes	CAT	SOD	APX	POX	Proline	Chlorophyll	RWC	Seedling shoot length	Seedling root length	Leaves number	Tillers number	Seedling shoot FW	Seedling shoot DW	Seedling root FW	Seedling root DW	Mature plant shoot DW	Mature plant root DW	Flag leaf length	Spikes number	Peduncle length	Spikes length	Healthy leaves percent	Rank
KC-29	9.5	8.5	11	8.5	9.5	1.5	2	8	3	9.5	5.5	8	6.5	11	8	3	2.5	5.5	2	1	6.5	1.5	6.19
KC-55	10	2	9.5	10.5	10	1.5	1.5	4.5	1	7.5	2.5	6	2.5	4.5	3	1	2	2	8	5.5	5.5	1	5.02
KC-58	5.5	8.5	9.5	6.5	4.5	1.5	1.5	2.5	2.5	6.5	4	5.5	1.5	11	3	8.5	7.5	4	9.5	8	7.5	8	5.75
KC-65	7.5	4	4	1.5	3.5	1.5	1.5	1.5	1	6.5	5.5	3	1	8.5	6	7	7.5	9	10	11	10	2	5.40
KC-82	9	7	7.5	8	5.5	2	1.5	8	2.5	1	3	1	2.5	6.5	4	5	3	9	10	11	10	3.5	5.58
KC-621	3.5	4	5.5	1.5	3.5	1	1.5	1	2	4	4	4	6.5	10	5	7	7.5	3	7	2.5	2	3	4.35
KC-839	3	6.5	1.5	6	1.5	1.5	1.5	4	2.5	6.5	5.5	1.5	3	11.5	7	7.5	5	5.5	9	1.5	6.5	6	4.71
KC-1749	8.5	7	6.5	5.5	7	1	1	1	2	4.5	2	4.5	10	2	2	7	4.5	9	10	11	10	2.5	5.63
KC-1772	2.5	8	9	3	8	1	1.5	7.5	3	5.5	3	2.5	6	3.5	2	7	9.5	8	3	6	7	2	5.13
KC-2009	8	3	7.5	2	7.5	1.5	1.5	4	1	2	2	9	8	6	4	3.5	4	9	10	11	10	2	5.54
KC-2015	3	3	2.5	1	2	1.5	1	7	4	10	6	3	6.5	7.5	4	4	6	8	4	7	9	1.5	4.88
KC-2115	7.5	4	9	4	2.5	1	1.5	4	2.5	7.5	3	5	9.5	13	7.5	10	9.5	4	9	8	1	2.5	5.88
KC-2120	3.5	9	8	10	8.5	1.5	1.5	2	3.5	8	3.5	4.5	7	14	8	5.5	6	4	9.5	4.5	7.5	4.5	6.04
KC-2121	4.5	3	4.5	7	1	1.5	1.5	4	3	9	4.5	5.5	8.5	9	5.5	6.5	7	1	9.5	9	1	5.5	5.02
KC-2122	3	4.5	3	7	7	1.5	1.5	2.5	2.5	8.5	2.5	5.5	5.5	13.5	4	1	1	4.5	5	3.5	6	7	4.52
KC-2123	7.5	7	6.5	2	6	1.5	1.5	2.5	3.5	7.5	2.5	8	1	13.5	8	5	6.5	3.5	6	10	3	6	5.23
KC-2189	2.5	3.5	1	7.5	2.5	1.5	1.5	5.5	2.5	9.5	5.5	4	3	5.5	2	4	7.5	6	9.5	8	9.5	7.5	4.73
KC-2225	**1.5**	**1**	**3**	**9.5**	**1.5**	**1.5**	**1.5**	**2.5**	**2.5**	**6**	**1.5**	**4.5**	**6.5**	**8.5**	**3.5**	**2**	**5.5**	**6**	**1**	**2**	**6.5**	**4**	**3.90**
KC-2226	**3.5**	**3.5**	**2**	**2**	**1.5**	**1.5**	**1.5**	**3**	**2.5**	**7.5**	**1**	**4**	**8**	**2.5**	**2**	**4**	**9**	**5.5**	**3**	**10**	**4.5**	**5.5**	**3.81**
KC-2231	**3**	**4**	**1**	**9**	**1**	**1.5**	**1.5**	**2.5**	**3.5**	**5**	**2**	**5.5**	**4**	**9.5**	**3**	**3.5**	**1**	**5**	**8**	**7.5**	**8.5**	**7**	**4.29**
KC-2241	1	2.5	3.5	5	2	1	1.5	6.5	2.5	2.5	2	6	6.5	8.5	4	4	5.5	5	8	9	5.5	6	4.40
KC-2248	6.5	5	9.5	12	2	1.5	1.5	2.5	3	5.5	2.5	5	2.5	12.5	4.5	6	8	7	2	9.5	3.5	7	5.25
KC-2286	2	5.5	9	11	2	1.5	1.5	3	2.5	3	1	8	1.5	1	1	9.5	10	5.5	7	10	1.5	7.5	4.52

Significant values are in bold.

### Morphological and physiological analysis of genotype KC-2226

According to Table 3, The RWC, seedling root length, and percentage of yellow leaves were decreased whereas seedling shoot length, the number of leaves and tillers, fresh and dry weight of roots and shoots as well as the percentage of tubular leaves were increased in the seedlings of genotype KC-2226 under drought stress. In mature plants, shoot dry weight and spikes number increased under drought stress, but root dry weight, and the length of flag leaf, peduncle, and spikes decreased (Table 3). In addition, under drought stress, proline content and activities of SOD, CAT, APX, and POX enzymes increased (Table 3).

**Table 3:** Mean comparison of morphological and physiological traits of KC-2226 genotype under drought stress.

Treatment	RWC (%)	Seedling shoot length (cm)	Seedling root length (cm)	Leaves number	Claws number	Seedling shoot FW (g)	Seedling root FW (g)	Seedling shoot DW (g)	Seedling root DW (g)	Yellow leaves (%)	Tubular leaves (%)
Control	74.19 ± 4.60	4.25 ± 0.14	36.5 ± 0.87	7.00 ± 0.58	2.00 ± 0.00	0.48 ± 0.00	1.45 ± 0.00	0.10 ± 0.00	0.15 ± 0.00	34.52 ± 2.49	0.00 ± 0.00
Drought stress	46.14 ± 5.00	6.50 ± 0.29	26.5 ± 3.75	22.67 ± 1.45	4.67 ± 0.33	1.53 ± 0.10	2.23 ± 0.09	0.45 ± 0.04	0.26 ± 0.01	29.63 ± 5.46	24.26 ± 2.28

Treatment	Mature plant shoot DW (g)	Mature plant root DW (g)	Flag leaf length (cm)	Peduncle length (cm)	Spikes length (cm)	Spikes number	Proline (μM/gFW)	SOD (Unit/mg protein)	CAT (μM H_2_O_2_ dec/min/mg protein)	APX (μM H_2_O_2_ dec/min/mg protein)	POX (μM H_2_O_2_ dec/min/mg protein)
Control	10.92 ± 0.47	7.75 ± 1.39	7.09 ± 0.05	3.00 ± 0.00	8.25 ± 0.14	5.67 ± 0.88	0.449 ± 0.001	0.077 ± 0.001	0.222 ± 0.001	0.089 ± 0.001	1.203 ± 0.002
Drought stress	13.17 ± 0.41	5.09 ± 0.33	5.92 ± 0.43	1.87 ± 0.40	6.89 ± 0.36	20.67 ± 0.33	3.287 ± 0.147	0.129 ± 0.017	0.435 ± 0.029	0.271 ± 0.016	3.425 ± 0.223

### Data quality and mapping

A total of 111,181,350 raw reads were constructed from two cDNA libraries. After trimming and removing the low-quality reads, a total of 95,042,354 clean reads were obtained (Table 4). A total of 89,089,860 reads were mapped to the genome with both forward and reverse primers, which included 93.75% of the total clean reads. A total of 3,792,499 reads (3.96% of clean reads) were mapped to genome with one either forward or reverse primer, and finally, a total of 2,159,995 reads (2.28% of clean reads) were not mapped to the genome (Table 4).

**Table 4:** The number of nucleotides and reads, mapped and unmapped reads with different primers in both control and drought treatment samples.

Sample	Total nucleotides	Total reads	Total nucleotides after trimming	Total reads after trimming	Reads mapped in pairs	Reads mapped in broken pairs	Reads not mapped
Control sample	8,060,123,340	55,779,076	6,068,287,661	45,129,426	42,449,472 (94.06%)	1,572,061 (3.48%)	1,107,893 (2.45%)
Drought stress sample	8,138,841,205	55,402,274	6,714,409,294	49,912,928	46,640,388 (93.44%)	2,220,438 (4.45%)	1,052,102 (2.11%)

### Differential gene expression analysis

**Figure 2:**
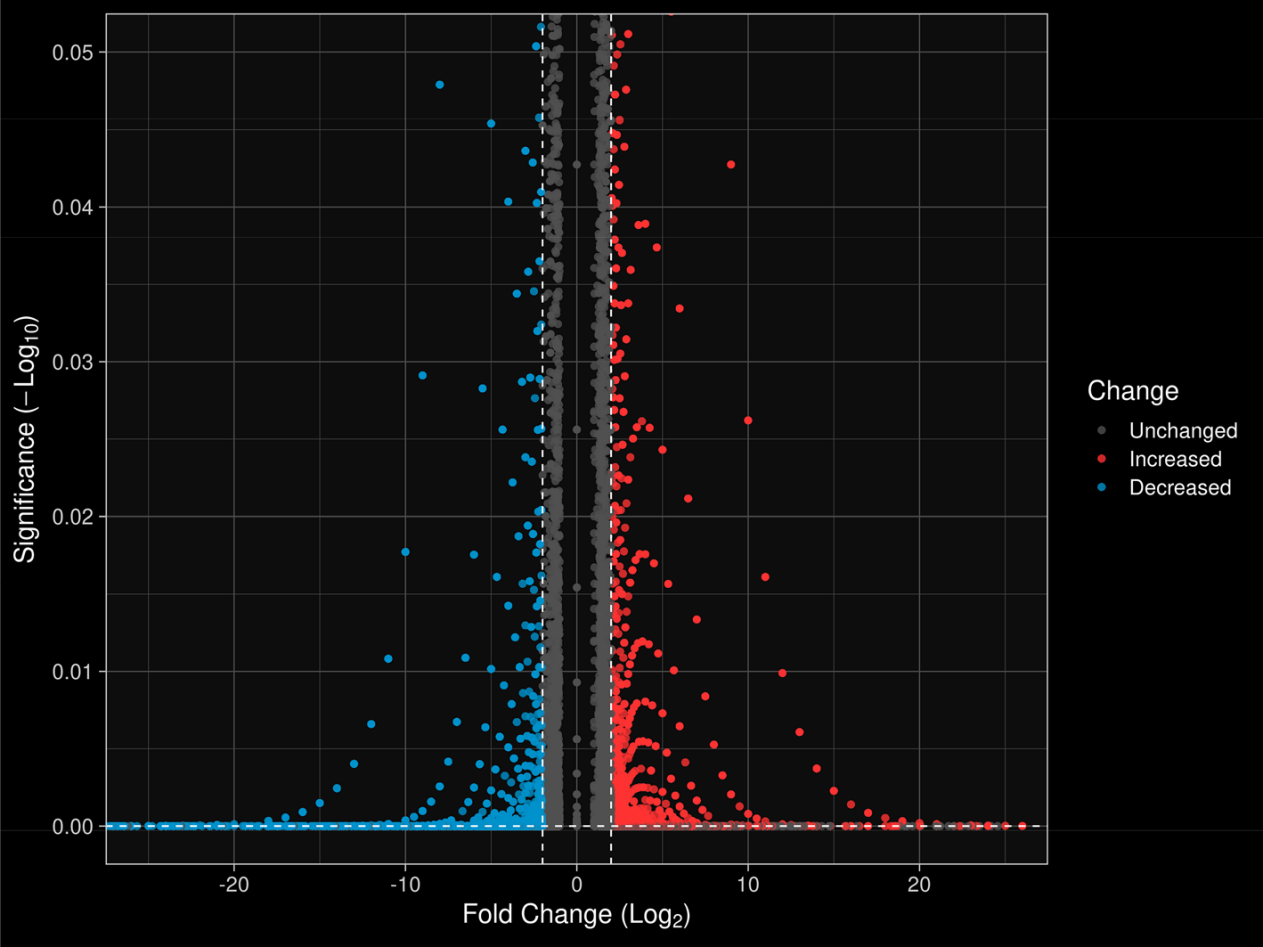
Distribution of differentially expressed genes (DEGs) under drought stress using volcano plot. Blue dots indicate down-regulated genes, red dots indicate up-regulated genes. The horizontal axis corresponds to the Logarithm Fold Change (LogFC) of genes. The vertical axis shows the level of significant probability (p-value) of the genes. Dashed lines separate the data with p-values below 0.05 as well as LogFC between + 2 and −2.

The expression of most genes varies between −10 ≤ LogFC ≤  + 10 (Fig. 2). According to the volcano plot, the distribution of upregulated and downregulated genes is almost similar. In this figure, the genes that were significant at the 0.05 probability level are shown. In total, 5007 genes were upregulated whereas 3489 genes showed a downregulation under drought stress (Fig. 2).

### Enrichment analysis

**Figure 3:**
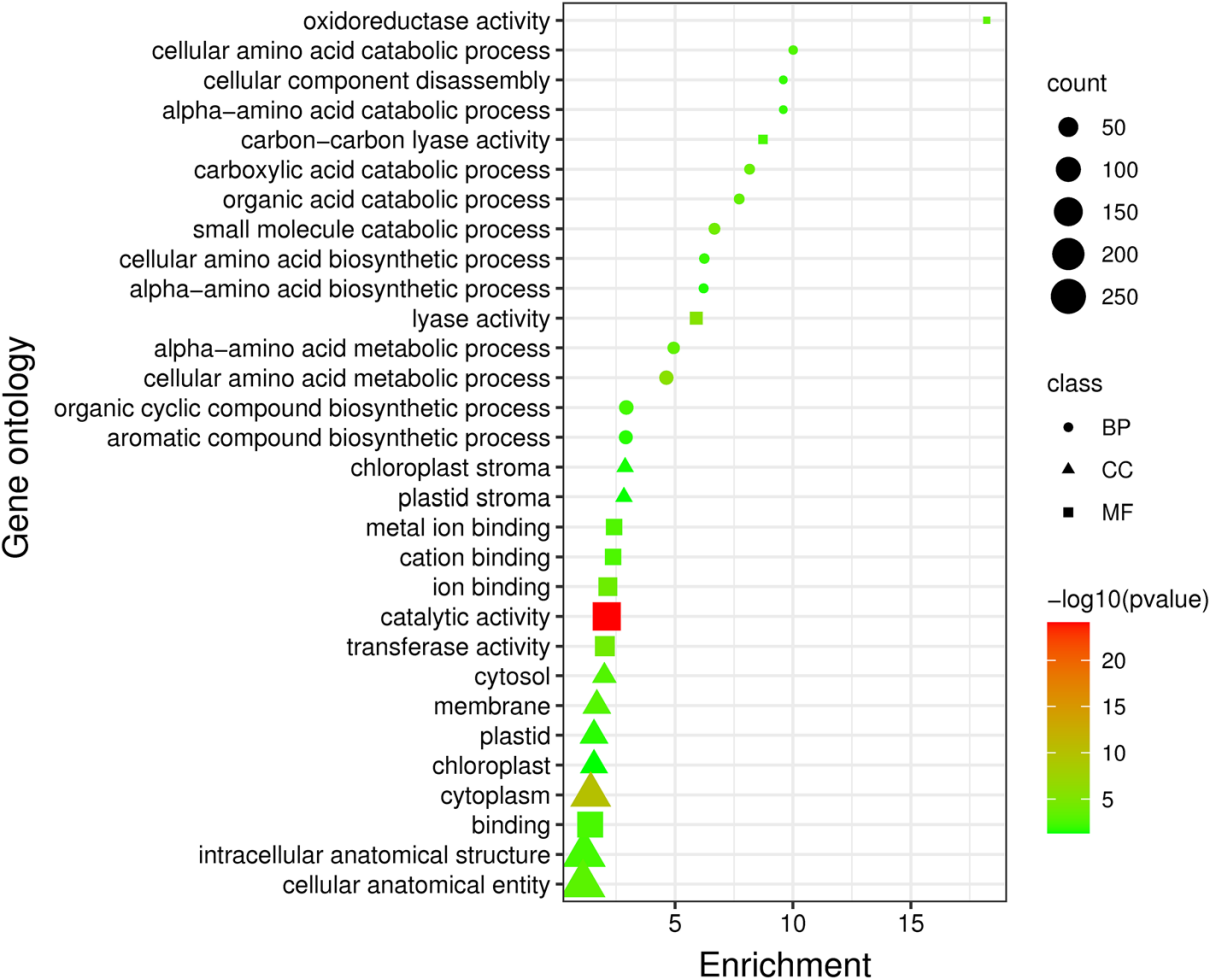
Gene ontology analysis of *Ae. tauschii* up-regulated genes under drought stress treatment vs control. Count: the number of genes involved in components of gene ontology classes. Class: the components of gene ontology include biological process, cellular component and molecular function. The color of the figures also shows the –Log10 (P-value), where the red color is the minimum P-value and the green color is the maximum P-value.

**Figure 4:**
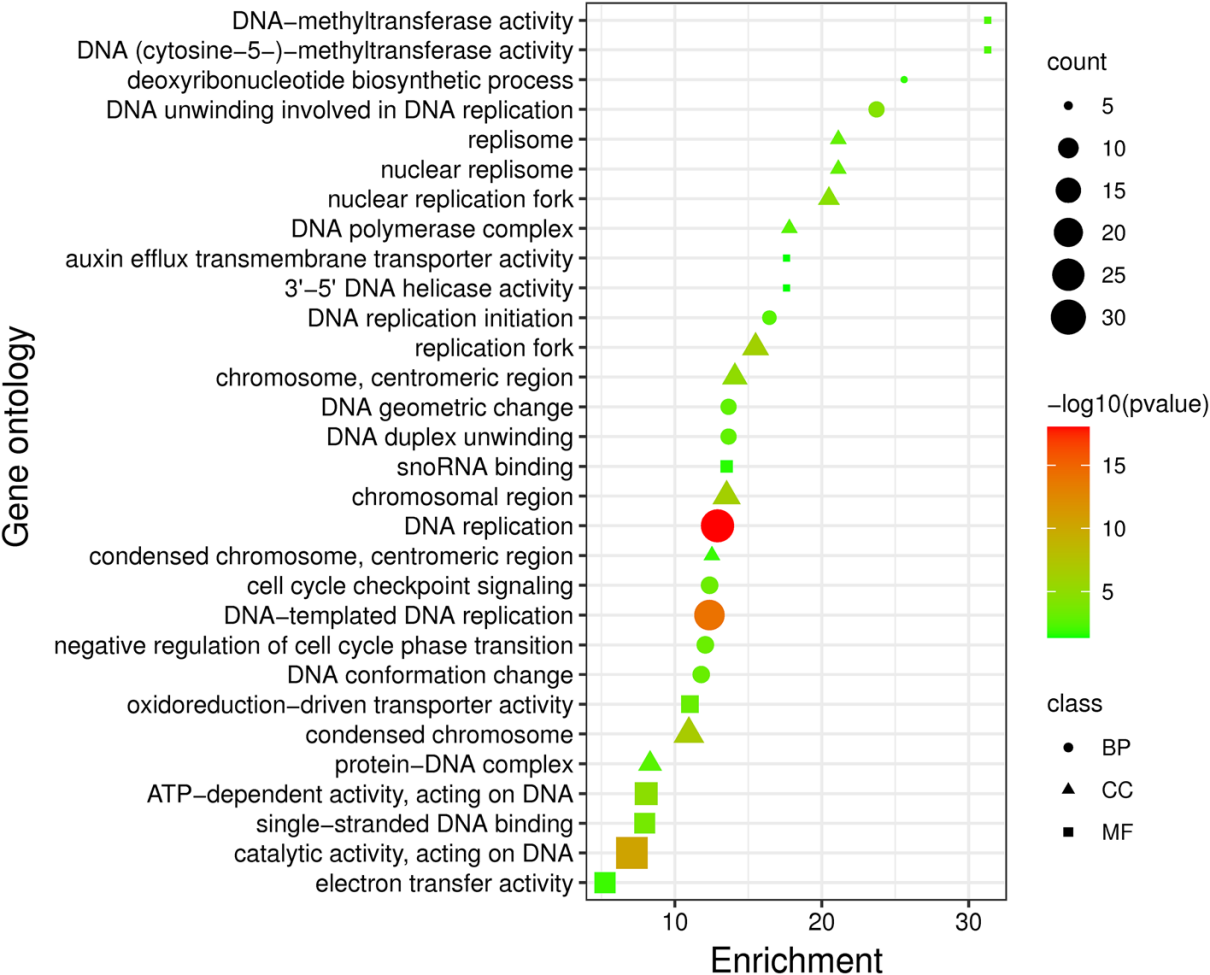
Gene ontology analysis of *Ae. tauschii* down-regulated genes under drought stress treatment vs control. Count: the number of genes involved in components of gene ontology classes. Class: the components of gene ontology include biological process, cellular component and molecular function.

Genes with enrichment values ≥ 1 were categorized into three classes of biological process, cellular component and molecular function (Figs. 3 and 4). Upregulated genes were often involved in biological activities of cellular component disassembly, carboxylic acid and organic acid catabolic process, cellular amino acid biosynthetic and metabolic process with enrichment values from 2.91 to 10.01, which were active in chloroplast, cytosol, and membrane. The molecular functions of these genes were mainly catalytic, lyase, and ion binding with enrichment values from 1.4 (cellular anatomical entity) to 18.22 (for oxidoreductase activity) (Fig. 3).

Downregulated genes were involved in biological processes of deoxyribonucleotide biosynthetic process, DNA unwinding cell cycle checkpoint signaling, and DNA conformation change with the enrichment values from 11.79 to 25.6. The deoxyribonucleotide biosynthetic process and DNA unwinding involved in DNA replication had the highest enrichment values (25.6 and 23.72, respectively).

These genes were active in nuclear replisomes, nuclear replication fork, DNA polymerase complex and chromosomes, which were involved in molecular functions including DNA-methyltransferase activity, transporter activity, DNA helicase activity and ATP-dependent activity with the enrichment values from 5.24 to 31.29. The DNA-methyltransferase activity had the highest enrichment value (31.29) in all gene ontology components (Fig. 4).

**Figure 5:**
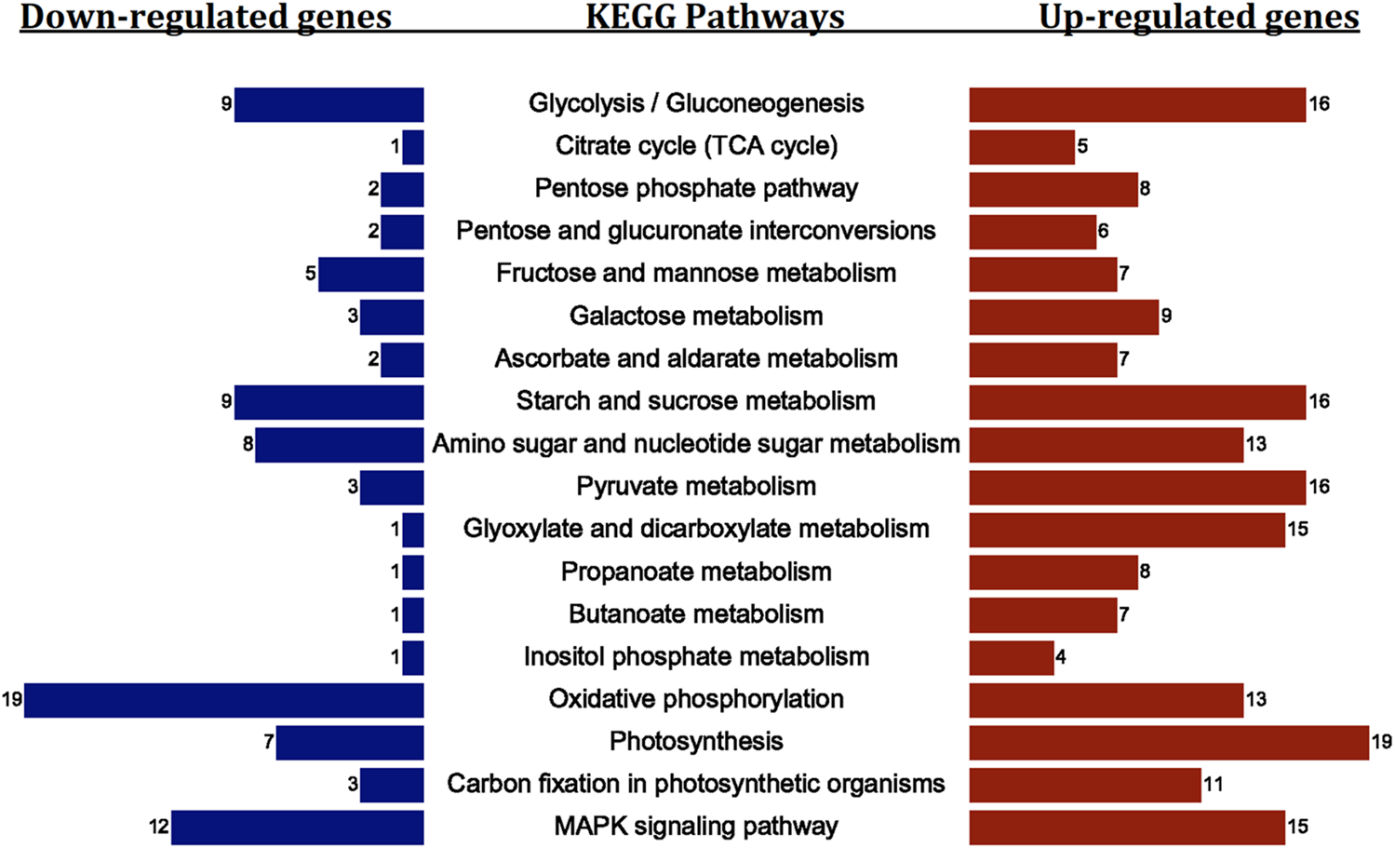
Important KEGG pathways of up- and downregulated genes under drought stress conditions in *Ae. tauschii*. The gene activity pathways that had a low P-value are in the middle, and the number of up- and down-regulated genes (as a bar plot) are also placed on the right (red) and left (blue) sides of the figure, respectively.

KEGG analysis was performed to annotate DEGs at the pathway level under drought stress (Fig. 5). Photosynthesis (19), glycolysis/gluconeogenesis (16), starch and sucrose metabolism (16), pyruvate metabolism (16), glyoxylate and dicarboxylate metabolism (15), and MAPK signaling pathway (15) had the highest number DEGs among upregulated genes. Oxidative phosphorylation (19), MAPK signaling pathway (12), glycolysis/gluconeogenesis (9), starch and sucrose metabolism (9), amino sugar and nucleotide sugar metabolism (8) and photosynthesis (7) were the pathways with highest number of downregulated genes.

**Figure 6:**
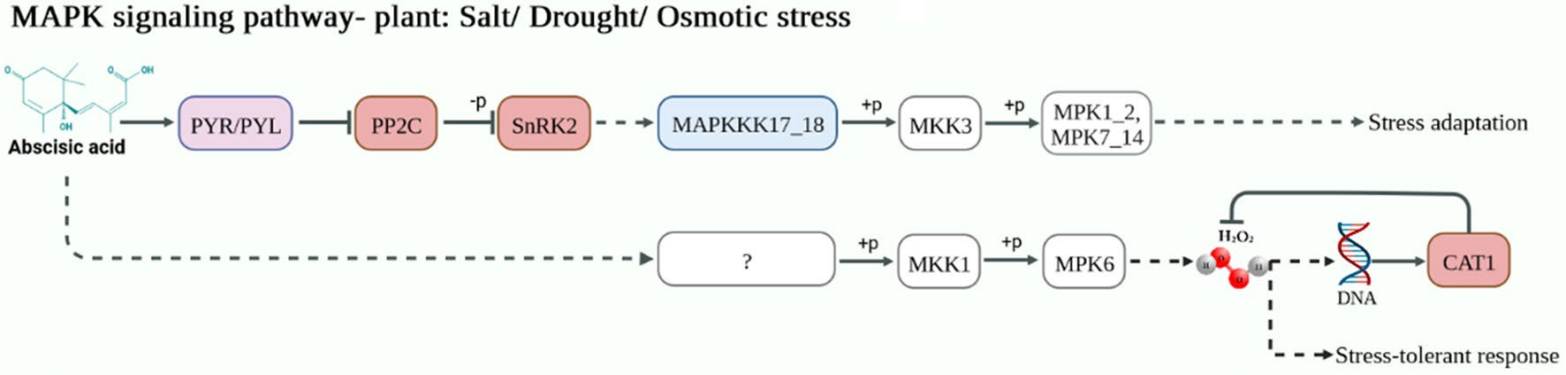
MAPK signaling pathway map of *A. tauschii* up- and downregulated genes under drought stress condition. The up-regulated genes are in red, the down-regulated genes are in blue, and the genes that were in both up- and down-regulated gene categories are in purple. Gene ID and LogFC of each gene are as follows: up-regulated: PYR/PYL (AET1Gv20314200: 2.79), PP2C (AET1Gv20867000: 1.83), SnRK2 (AET2Gv21287400: 1.59), CAT1 (AET6Gv20106200l: 5.68). Down-regulated: PYR/PYL (AET4Gv20498300: −3.2), MAPKKK17-18 (AET3Gv20622200: −12.5).

Our result showed that MAPK signaling pathway is one of important active pathways involved in drought stress conditions (Fig. 6). Once stress signals were perceived by cell membranes, this pathway is regulated by PRY/PRL as soluble abscisic acid (ABA) receptors in an ABA-dependent way. The 2C-type protein phosphatases (PP2C), which inactivates SNF1-related protein kinases2 (SnRK2) through dephosphorylation, is inhibited in the presence of ABA. The activated SnRK2, in biotic stress such as drought and salinity, triggers plant responses and regulates transcriptional factors to synthesize functional proteins (FP). Second: in a classical MAPK signaling cascade, MAPKKK is activated by stimulated plasma membrane receptors and transmits signals downstream. MAPKKK activates MAPKK by phosphorylating the conserved S/T-XXXXX-S/T motif in MAPKK. Subsequently, MAPKK activates MAPK by phosphorylating the TXY motif in MAPK. Finally, MAPK activates downstream kinases, enzymes, transcription factors and other response factors and transmits extracellular environmental signals into cells.

### PPI network

The summary of PPI analysis is listed in Table 5. The number of nodes and edges were approximately two and eight times, respectively, more in PPI network of downregulated genes compared with those in upregulated genes. The average local clustering coefficient of PPI network of up- and downregulated genes were 0.332 and 0.41, respectively, with the same P-value.

**Table 5:** String PPI Information of up- and down-regulated genes in *Ae. tauschii* under drought stress conditions.

PPI information	Upregulated	Downregulated
Number of nodes	287	511
Number of edges	542	4364
Average node degree	3.78	17.1
Average local clustering coefficient	0.332	0.41
Expected number of edges	330	2111
PPI enrichment p-value	< 1.0e−16	< 1.0e−16

**Figure 7:**
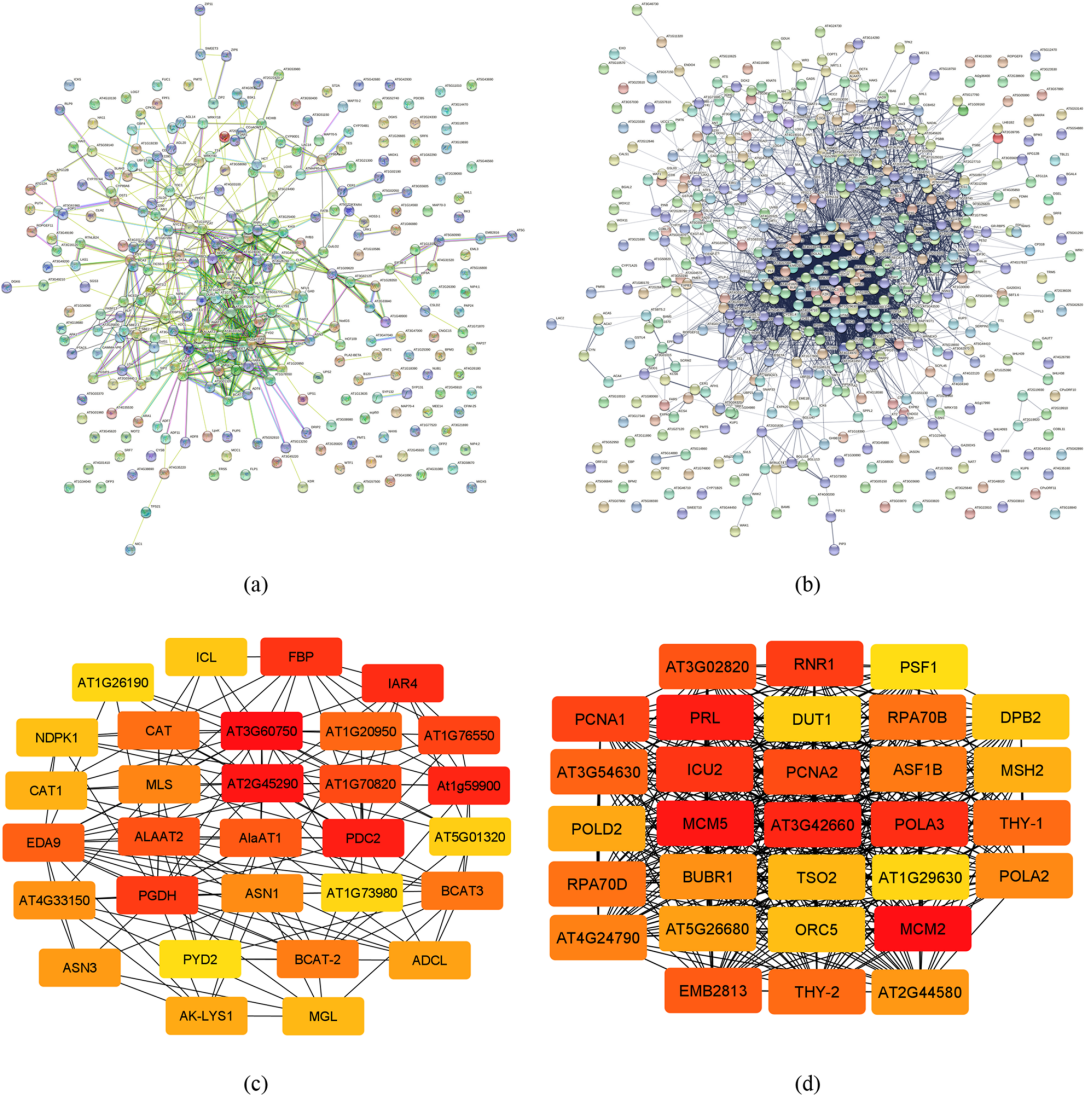
String PPI network and CytoHubba gene network of up- and downregulated genes in *A. tauschii* under drought stress conditions. (**a**) PPI network of upregulated genes. (**b**) PPI network of downregulated genes. (**c**) Gene network of upregulated genes. (**d**) Gene network of downregulated genes. In gene networks, the color intensity determines the rank of the genes, the higher the color intensity and closer to red, the higher the score, and the closer to yellow, the lower the score. Genes with higher scores are more important to us and are our hub genes.

Gene network was constructed through the data including nodes and edges acquired from PPI analysis (Fig. 7). Genes pyrophosphate-fructose 6-phosphate 1-phosphotransferase subunit alpha: PFP-ALPHA (1.46), PFPA1 (1.42), IAA-conjugate-resistant 4 (IAR4) (1.19), Dihydropyrimidinase (PYD2) (1.16), TTM2 (1.14), TTM1 (1.14), SYYC2 (1.13), SR543 (1.13), and SYYC1 (1.13) among upregulated genes and bifunctional dihydrofolate reductase-thymidylate synthase 2 (THY-2) (44), proliferating cellular nuclear antigen 1 (PCNA1) (41), DNA topoisomerase 2 (TOPII) (22), emb2411 (18), NIA1 (18), THY-1 (16) and RNR1 (15) among downregulated genes had the highest scores and recognized as hub genes (Fig. 7).

### Expression analysis and validation of RNA-seq data

**Figure 8:**
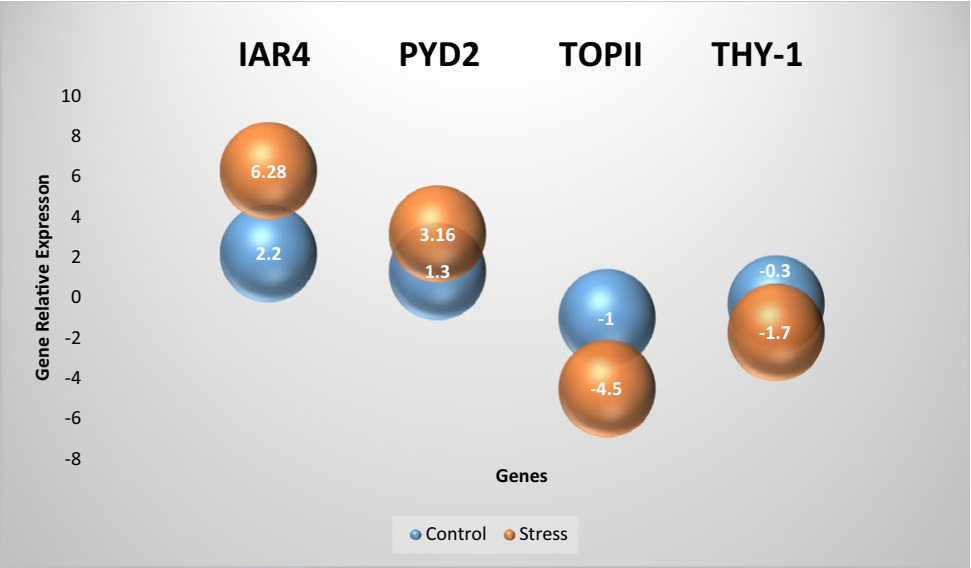
Expression analysis of hub genes in drought stress treatment (orange color) and control (blue color).

The expression level of two upregulated genes IAR4 and PYD2, and two downregulated genes TOPII and THY-1 under control and drought stress treatments were analyzed and then compared with RNA-seq results. The expression of IAR4 and PYD2 genes increased under the control and stress treatments with relatively higher expression in the drought stress treatment than those in the control. The expression of IAR4 (6.28) and PYD2 (3.16) under stress treatment were almost three times more than their control (2.2 and 1.3, respectively). The expression of TOPII and THY-1 genes decreased under control conditions and under drought stress. The severity of the decrease in the expression of these two genes in drought stress conditions was significantly higher than in the control. The expression of TOPII gene under drought stress decreased by 4.5 times compared to the control condition, while the expression of THY-1 gene decreased by 5.6 times under drought stress compared to the control condition (Fig. 8). The RT-qPCR result for selected genes was almost consistent with the RNA-seq data. LogFC of IAR4, PYD2, TOPII and THY-1 genes were 13.94, 4.54, −4.2 and −3.58, respectively.

## Discussion

Since the DD genome of *Ae. tauschii* Coss is a good source of resistance to different biotic and abiotic stresses, resistance genes from this species can be transferred to bread wheat^16^ or its close relatives through classical approaches for breeding purposes and increasing the quality of wheat cultivars^5^. To find the most important genes involved in drought stress, at first, genotype KC-2226 was selected among 23 other Iranian *Ae. tauschii* genotypes as the most tolerant one according to morphological and physiological properties.

Transcriptomic analysis of genotype KC-2226 was carried out and DEGs were identified under drought stress. PPI analysis was done and the gene network was constructed using CytoHubba by employing MCC method, which had a better performance on the prediction accuracy of important proteins from the PPI network. The most important upregulated genes were AT1G76550, AT1G20950, IAR4, and PYD2 whereas the most important downregulated genes included THY-2, PCNA1, and TOPII (Fig. 7).

Genes AT1G76550 and AT1G20950 are involved in the process of photosynthesis and response to glucose. These genes are located in cytoplasm and mitochondria and are a part of pyrophosphate-dependent phosphofructokinase complex and alpha-subunit complex. These genes are active in the glycolysis/gluconeogenesis, fructose and mannose metabolism and pentose phosphate pathways^33^, having molecular functions including ATP binding, phosphofructokinase, and diphosphate-fructose-6-phosphate 1-phosphotransferase activity^34^.

The PFPs are involved in response to different stresses including dehydration, high salt, phosphate starvation anoxia and wounding^35,36^. They play roles in gluconeogenesis, glycolysis, stabilization of triose-phosphate and hexose-phosphate pools, and regulation of inorganic pyrophosphate (PPi) concentration during synthesis and degradation of sucrose as well as adaptability to stresses^36–38^. Lim et al. (2014) investigated the response of PFP double and quadruple knockout mutants to osmotic and salt stresses to clarify the role of PFP in the stress tolerance of Arabidopsis seedlings. The expression of PFP subunit genes increased in response to salt and osmotic stresses. These findings suggest that PFP plays a role in adapting to salt and osmotic stresses^39^.

The IAR4 is involved in the biosynthesis of acetyl-CoA from pyruvate, glycolytic processes as well as auxin conjugate sensitivity and homeostasis in root development^40^. This gene is located in the cytosol, mitochondria, and mitochondrial matrix with pyruvate dehydrogenase (acetyl-transferring), and cobalt and zinc ion binding activities^41^. It is engaged in glycolysis/gluconeogenesis, pyruvate and carbon metabolism and citrate cycle pathways^33^. Fu et al. (2019) reported that IAA-CONJUGATE-RESISTANT 4 (IAR4) plays a key role in primary root growth under salt stress conditions. Mutation of IAR4 led to increased sensitivity to salt stress conditions, with strongly inhibited primary root growth and reduced survival rate in two iar4 mutant alleles^42^.

The PYD2 is involved in the beta-alanine biosynthetic process, cellular response to nitrogen levels, pyrimidine nucleobase catabolic process, and uracil catabolic process. The PYD2 is located in endomembrane system, endoplasmic reticulum, Golgi apparatus, and plastid with dihydropyrimidinase activity and metal ion binding activities. It is engaged in amino acid and beta-alanine biosynthesis pathways.

The THY-1 is involved in the biosynthesis of 10-formyltetrahydrofolate and dTMP, methylation and one-carbon metabolic process. This gene is in cytosol and mitochondria with dihydrofolate reductase and thymidylate synthase activities, which are crucial for DNA synthesis. These two enzymatic activities in plants are expressed as one bifunctional enzyme^43^. In addition, it is active in pathways of cofactor biosynthesis and tetrahydrofolate biosynthesis. Gorelova et al. (2017) showed that one of the DHFR-TS (dihydrofolate reductase-thymidylate synthase) isoforms (DHFR-TS3) operates as an inhibitor of its two homologs, thus regulating DHFR and TS activities and, as a consequence, folate abundance. In addition, a novel function of folate metabolism in plants is proposed, i.e., maintenance of the redox balance by contributing to NADPH production through the reaction catalyzed by methylenetetrahydrofolate dehydrogenase, thus allowing plants to cope with oxidative stress^44^.

The PCNA1 is involved in the biological elongation of the leading strand, repair of mismatch, regulation of DNA replication, regulation of cell cycle and translation. It plays different functions including DNA binding, DNA polymerase processivity factor activity in the cytoplasm, cytosol, nucleolus, nucleus and PCNA complex. The PCNA1 is active in DNA replication, base and nucleotide excision and mismatch repair, cell cycle^45^. Ghabooli et al. (2013) conducted a proteomics study to understand the molecular mechanisms underlying water stress tolerance induced by *Piriformospora indica* in barley. They reported that the abundance of PCNA decreased in response to drought stress. However, *P. indica* colonization resulted in an increase in the abundance of this protein under drought conditions^46^.

The TOPII is engaged in DNA topological change and as an active cellular component of intracellular membrane-bounded organelle has different roles including ATP binding and hydrolyzing, metal ion binding, DNA binding, and double-strand break. Topoisomerases mitigate topological stress by untangling and relaxing the supercoiled DNA in both eukaryotes and prokaryotes^47,48^. John et al. (2016) over-expressed topoisomerase II (TopoII) in tobacco (*Nicotiana tabaccum*) and examined its role in growth and development as well as salt (NaCl) stress tolerance. They revealed that NtTopoII1-α over-expression in tobacco confers salt stress tolerance to the transformed lines as compared to wild-type plants. TopoII over-expression changed the morphology of the transgenic plants and improved the seed germination on a salt-supplemented medium^49^.

Altogether, upregulated genes were often involved in photosynthesis, glycolysis and gluconeogenesis, amino acid and beta-alanine biosynthesis whereas downregulated genes were mainly involved in DNA synthesis, replication, repair and topological changes. These results indicated that photosynthesis, glycolysis, and gluconeogenesis are prioritized in *Ae. tauschii* grown under drought stress to provide more energy for the plant than DNA-related activities. Still, understanding the mechanism of the most important pathways dealing with drought stress needs further studies.

## Conclusion

*Ae. tauschii* is known as a plant tolerant to all kinds of biotic and abiotic stresses. Therefore, identifying the genes with the highest interaction, both up- and downregulated genes, under drought stress is of prime importance. According to our results, the plant exploits transcription of specific genes (photosynthesis, glycolysis and gluconeogenesis, amino acid, and beta-alanine biosynthesis pathways) instead of DNA synthesis and repair under stress conditions to provide the energy needed for the plant to survive under stressful conditions.

## Supplementary Information

Supplementary Information.

## Data Availability

The datasets generated during the current study are available in the NCBI database, BioProject ID of PRJNA868361.
