# Supplementary material for: Identification of drought-tolerant hub genes in Iranian KC-2226 genotype of Aegilops tauschii using transcriptomic analysis
Source: Sci Rep. 2023 Jun 12;13:9499. doi: 10.1038/s41598-023-36133-0 (PMC10261118; doi:10.1038/s41598-023-36133-0)
Supplement: Supplementary file 1 — Supplementary Information. [file 41598_2023_36133_MOESM1_ESM.docx]

Supplementary file 1. Genotype code and collection place of *Ae. tauschii* genotypes in this experiment.

| No. | Genotype code | Accusable  Gene Bank /institute | WIEWS inst.code | Country | Province | Location |
| --- | --- | --- | --- | --- | --- | --- |
| 1 | KC-29 | IUGB | 00279 | Iran | East Azerbaijan | 10 km of Ahar-Tabriz road |
| 2 | KC-55 | IUGB | 00362 | Armenia | - | - |
| 3 | KC-58 | IUGB | 00367 | Iran | East Azerbaijan | 10 km of Ahar-Tabriz road |
| 4 | KC-65 | IUGB | 00386 | Iran | East Azerbaijan | 10 km of Ahar-Kaleybar road |
| 5 | KC-82 | NPGBI | - | Iran | Gilan | Rasht |
| 6 | KC-621 | NPGBI | IRN029 | Iran | North Khorasan | Bojnurd |
| 7 | KC-839 | NPGBI | IRN029 | Iran | Mazandaran | Sari |
| 8 | KC-1749 | NPGBI | IRN029 | Iran | Semnan | Shahroud |
| 9 | KC-1772 | NPGBI | IRN029 | Iran | Mazandaran | - |
| 10 | KC-2009 | NPGBI | IRN029 | Iran | Mazandaran | Behshahr |
| 11 | KC-2015 | NPGBI | IRN029 | Iran | Mazandaran | Nur |
| 12 | KC-2115 | NPGBI | IRN029 | Iran | Razavi Khorasan | Mashhad |
| 13 | KC-2120 | NPGBI | IRN029 | Iran | North Khorasan | Bojnurd |
| 14 | KC-2121 | NPGBI | IRN029 | Iran | North Khorasan | Bojnurd |
| 15 | KC-2122 | NPGBI | IRN029 | Iran | North Khorasan | Bojnurd |
| 16 | KC-2123 | NPGBI | IRN029 | Iran | North Khorasan | Bojnurd |
| 17 | KC-2189 | NPGBI | IRN029 | Iran | Qazvin | Qazvin |
| 18 | KC-2225 | NPGBI | IRN029 | Iran | Ardabil | Khalkhal |
| 19 | KC-2226 | NPGBI | IRN029 | Iran | Ardabil | Khalkhal |
| 20 | KC-2231 | NPGBI | IRN029 | Iran | Ardabil | Khalkhal |
| 21 | KC-2241 | NPGBI | IRN029 | Iran | Ardabil | Khalkhal |
| 22 | KC-2248 | NPGBI | IRN029 | Iran | Ardabil | Khalkhal |
| 23 | KC-2286 | NPGBI | IRN029 | Iran | Chaharmahal and Bakhtiari | Boroujen |
